# Efficacy of the 'Five-Needle' method for pancreatojejunostomy in laparoscopic pancreaticoduodenectomy: an observational study

**DOI:** 10.3389/fonc.2024.1347752

**Published:** 2024-04-16

**Authors:** Zheng-Feng Wang, Bo Zhang, Hao Xu, Wen-Ce Zhou

**Affiliations:** ^1^ The Fourth Ward of General Surgery, The First Hospital of Lanzhou University, Lanzhou, China; ^2^ The Second Hospital of Lanzhou University, Lanzhou, China

**Keywords:** laparoscopic pancreaticoduodenectomy (LPD), Five-Needle method, duct-to-mucosa method, pancreatic fistula, operative time

## Abstract

**Objective:**

The five-needle pancreato-intestinal anastomosis method is used in laparoscopic pancreaticoduodenectomy (LPD). The aim of this study was to explore the clinical efficacy and adverse reactions of this new surgical method and to provide a scientific reference for promoting this new surgical method in the future.

**Methods:**

A single-centre observational study was conducted to evaluate the safety and practicality of the five-needle method for pancreatojejunostomy in LPD surgeries. The clinical data of 78 patients who were diagnosed with periampullary malignancies and underwent LPD were collected from the 1^st^ of August 2020 to the 31^st^ of June 2023 at Lanzhou University First Hospital. Forty-three patients were treated with the ‘Five-Needle’ method (test groups), and 35 patients were treated with the ‘Duct-to-Mucosa’ method (control group) for pancreatojejunostomy. These two methods are the most commonly used and highly preferred pancreatointestinal anastomosis methods worldwide. The primary outcome was pancreatic fistula, and the incidence of which was compared between the two groups.

**Results:**

The incidence of pancreatic fistula in the five-needle method group and the duct-to-mucosa method group was not significantly different (25.6% vs. 28.6%, p=0.767). Additionally, there were no significant differences between the two groups in terms of intraoperative blood loss (Z=-1.330, p=0.183), postoperative haemorrhage rates (p=0.998), length of postoperative hospital stay (Z=-0.714, p=0.475), bile leakage rate (p=0.745), or perioperative mortality rate (p=0.999). However, the operative time in the ‘Five-Needle’ method group was significantly shorter than that in the ‘Duct-to-Mucosa’ method group (270 ± 170 mins vs. 300 ± 210 mins, Z=-2.336, p=0.019). Further analysis revealed that in patients with pancreatic ducts smaller than 3 mm, the incidence of pancreatic fistula was lower for the ‘Five-Needle’ method than for the ‘Duct-to-Mucosa’ method (12.5% vs. 53.8%, p=0.007).

**Conclusion:**

The five-needle method is safe and efficient for pancreatojejunostomy in LPD, and is particularly suitable for anastomosis in nondilated pancreatic ducts. It is a promising, valuable, and recommendable surgical method worthy of wider adoption.

## 1 Introduction

Laparoscopic pancreaticoduodenectomy (LPD), as one of the more complex general surgical procedures, remains the primary surgical method for treating periampullary malignancies. The procedure, involving concomitant organ resection and the establishment of gastroenteric, hepaticojejunostomy, and pancreatojejunostomy anastomoses, is associated with postoperative complications such as pancreatic fistula, bile leakage, intra-abdominal haemorrhage, and infection, posing significant postoperative risks to patients (1–3). Clinical studies indicate that the incidence of postoperative pancreatic fistula ranges from 21.4% to 28.0%, with severe fistulas (grades B and C) occurring in approximately 8% and 12.2%, respectively, of patients experiencing postoperative haemorrhage. The surgery-related mortality rate is reported to be between 3% and 6% (1–3). Inadequate healing of the pancreatojejunostomy is a primary contributor to these complications. Clinical manifestations include not only pancreatic fistulas but also leakage of pancreatic fluid rich in amylase, lipase, and protease, increasing the risk of secondary complications such as intra-abdominal haemorrhage and infection (4, 5).

Since the first LPD reported by Gagner et al. in 1994 (6), minimally invasive methods have been applied to pancreaticoduodenectomy, especially with recent advancements in laparoscopic and robotic-assisted technologies. Various LPD methods have been proposed, somewhat simplifying the anastomosis process (7, 8). However, the rate of postoperative complications has not significantly decreased, remaining a major hindrance to the successful execution of laparoscopic pancreatic surgery (9–11). Therefore, an LPD method that is not only straightforward in terms of execution but is also effective in reducing the incidence of pancreatic fistula and bleeding is urgently needed.

Open and LPD surgeries were first introduced 20 years ago, and due to an improved understanding of tissue healing physiology and the specifics of laparoscopic surgical methods, we have developed an innovative, straightforward, safe, and effective method for LPD, termed the five-needle method. The aim of this study was to preliminarily evaluate the clinical effectiveness and application value of the five-needle method for pancreatojejunostomy in LPD.

## 2 Materials and methods

### 2.1 Patients

This single-centre retrospective study was conducted to evaluate the safety and practicality of the five-needle method for pancreatojejunostomy in LPD. The clinical data of 116 patients who were diagnosed with periampullary malignancies and underwent LPD were collected from the 1^st^ of August 2020 to the 31^st^ of June 2023 at Lanzhou University First Hospital. After excluding 38 patients (18 with benign pathology diagnosed postoperatively without regional lymph node dissection, 14 who required conversion to open surgery, and 6 with incomplete observational data), 78 patients were enrolled in the study. Forty-three patients were treated with the ‘Five-Needle’ method (test groups), and 35 patients were treated with the ‘Duct-to-Mucosa’ method (control group) for pancreatojejunostomy. These two methods are the most commonly used and most highly preferred pancreatointestinal anastomosis methods worldwide.

### 2.2 Intervention measures

#### 2.2.1 'Five-Needle' method

**Figure 1:**
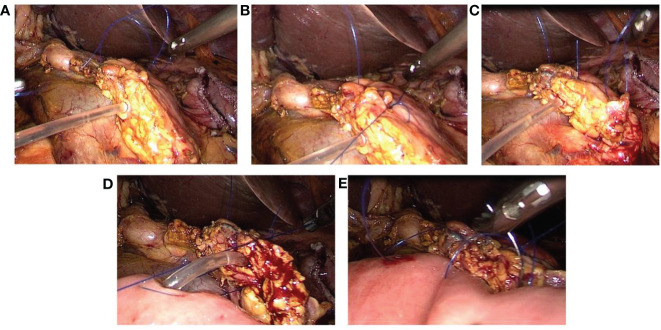
Laparoscopic ‘Five-Needle’ method for Pancreatojejunostomy. **(A)** A silicone tube is inserted into the main pancreatic duct, followed by a ‘U’-shaped suture through the upper margin of the pancreatic stump using 4-0 Prolene (the first needle). **(B)** A 4-0 Prolene suture is passed through the upper and lower edges of the pancreatic duct and used for a ‘U’-shaped suture through the middle of the pancreatic stump (the second needle). **(C)** A ‘U’-shaped suture through the lower margin of the pancreatic stump is executed with 4-0 Prolene (the third needle). **(D)** A small opening is made on the opposite side of the jejunal mesentery, and a 4-0 Prolene suture is used for a ‘U’-shaped pancreatojejunostomy. The pancreatic stent tube is inserted into the jejunum without tying the suture immediately (the fourth needle). **(E)** A continuous suture through the full thickness of the pancreatic stump and the seromuscular layer of the jejunum is performed with 4-0 Prolene, from the upper to the lower margin of the pancreas. The ends of this suture are tied to the reserved ends of the first and third sutures (the fifth needle).

- A silicone tube matching the internal diameter of the main pancreatic duct is inserted through the cut end of the duct, leaving approximately 3-4 cm of the tube extending beyond the pancreatic remnant.
- Three 4-0 Prolene ‘U’-shaped interrupted sutures are stitched through the end of the pancreas. These sutures close the branch pancreatic ducts, ensure haemostasis, and firmly fix the stent tube to the main pancreatic duct to prevent dislodgement. The upper and lower sutures are tied, leaving approximately 4 cm of suture tail for later use (**Figures 1A–C**).
- The jejunum is brought up adjacent to the pancreatic remnant. A small opening is created on the antimesenteric border of the jejunum at an appropriate location. The fourth 4-0 Prolene vascular suture is used for a “U”-shaped pancreatojejunostomy. Both ends of the suture pass through the front of the pancreas, entering and exiting. The pancreatic stent tube is inserted into the jejunum, which is closed but not tied, to facilitate the fifth suture (**Figure 1D**).
- The fifth 4-0 Prolene vascular suture creates a continuous full-thickness anastomosis from the upper to the lower edge of the pancreatic stump and the seromuscular layer of the jejunum. The fourth suture is then tightened and tied, followed by tightening the fifth suture. The ends of this suture are tied to the residual ends of the first and third sutures (**Figure 1E**).

#### 2.2.2 ‘Duct-to-Mucosa’ method

**Figure 2:**
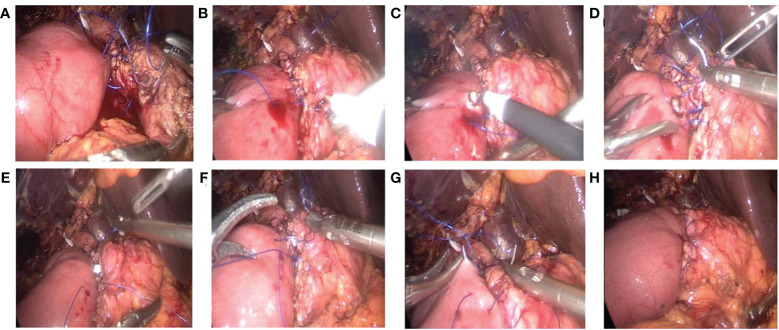
Laparoscopic ‘Duct-to-Mucosa’ pancreatojejunostomy. **(A)** Continuous suture of the posterior wall of the pancreatic stump to the jejunum using 4-0 Prolene. **(B)** The suture line is tightened upon completion of the posterior wall suturing. **(C)** A small opening is created in the jejunum with an electrocautery hook. **(D)** Continuous suturing of the posterior wall of the pancreatic duct to the jejunal opening using 5-0 Prolene. **(E, F)** After the insertion of the pancreatic duct stent tube, the anterior wall of the pancreatic duct and the jejunal opening are sutured together using 5-0 Prolene. **(G, H)** The anterior wall of the pancreatic stump is continuously sutured to the jejunum using 4-0 Prolene.

- The neck of the pancreas is adequately mobilized to expose the cut end of the pancreatic duct. Continuous suturing of the posterior wall of the pancreatic stump to the seromuscular layer of the intestine is performed using 4-0 Prolene sutures from the upper to the lower margin of the pancreatic stump, followed by tightening the suture (**Figures 2A, B**).
- A small opening corresponding to the diameter of the pancreatic duct is created on the antimesenteric border of the jejunum (near the cut end of the pancreatic duct) using an electrocautery hook (**Figure 2C**).
- The posterior wall of the pancreatic duct and the posterior wall of the jejunal opening are continuously sutured together using 5-0 Prolene sutures. A pancreatic duct stent tube, matching the diameter of the pancreatic duct, is inserted, with one end of the stent placed into the jejunal lumen. The anterior wall of the pancreatic duct and the anterior wall of the jejunal opening are then continuously sutured together with the same 5-0 Prolene sutures, completing the continuous ‘Duct-to-Mucosa anastomosis’ (**Figures 2D–F**).
- The anterior wall of the pancreatic stump is continuously sutured to the seromuscular layer of the intestine using the original 4-0 Prolene sutures from the lower to the upper margin of the pancreatic stump, followed by tightening the suture to complete the pancreatojejunostomy (**Figures 2G, H**).

### 2.3 Outcome measures

#### 2.3.1 Primary outcome indicators

Pancreatic Fistula: Postoperative pancreatic fistulas are characterized the presence of an amylase concentration in the abdominal cavity drainage fluid that is more than three times the upper limit of normal serum amylase levels, coupled with relevant clinical symptoms necessitating active intervention. Based on the 2016 classification scheme of the International Study Group on Pancreatic Fistula (ISGPF) (12), fistulas are classified as Grade A (biochemical leak), Grade B, or Grade C (**Table 1**). Grade B or C fistulas are categorized as severe pancreatic fistulas.

**Table 1:** Classification of pancreatic fistula.

Grade	Grade A	Grade B	Grade C
The concentration of amylase in drainage fluid was 3 times higher than the upper limit of amylase in serum	Yes	Yes	Yes
Continuous peripancreatic drainage ≥ 3 weeks	No	Yes	Yes
Changes in clinical decision-making related to pancreatic fistula	No	Yes	Yes
The effusion needs to be resolved by puncture drainage	No	Yes	Yes
Pancreatic fistula-associated haemorrhage was studied by angiography	No	Yes	Yes
Signs of pancreatic fistula associated infection	No	Yes (not associated with organ failure)	Yes (not associated with organ failure)
Reoperation	No	No	Yes
Pancreatic fistula-related organ failure	No	No	Yes
Pancreatic fistula-related death	No	No	Yes

#### 2.3.2 Secondary outcome indicators

Operative time: The duration from the completion of anaesthesia and related preparations to the establishment of pneumoperitoneum until the closure of the abdomen.

Intraoperative blood loss Amount of blood loss recorded in the surgical records.

Postoperative hospital stay The time from the end of the surgery to the patient’s discharge (if the patient underwent a second surgery due to postoperative complications, the duration was considered the end of the first surgery).

Bile leak: A bile concentration in the abdominal drainage fluid or ascites more than three times the upper limit of normal serum bilirubin levels, persisting for more than 3 days postoperatively, or requiring interventional treatment or reoperation due to bile accumulation or biliary peritonitis.

Postoperative haemorrhage: Postoperative bleeding manifested through abdominal drainage tubes and/or gastrointestinal decompression tubes, possibly presenting as rectal bleeding, accompanied by symptoms of haemorrhagic shock such as hypotension and tachycardia and a decrease in haemoglobin concentration.

Perioperative mortality: Death of a patient during surgery or during the postoperative hospital stay due to surgery-related complications or cardiovascular incidents induced by the surgery.

### 2.4 Inclusion and exclusion criteria

The study included a postoperative pathological diagnosis of periampullary malignancies (cholangiocarcinoma, pancreatic head cancer, duodenal papillary cancer) and complete surgical data. The exclusion criteria were as follows: 1) a postoperative pathological diagnosis of benign periampullary disease; and 2) Incomplete laparoscopic surgery.

### 2.5 Data collection and handling

A standardized protocol was implemented to maintain consistency in interviewer training and quality control supervision across all instances of data collection. Trained interviewers utilized a standardized questionnaire to gather the following information: age, sex, body mass index (BMI), pancreatic duct diameter, and pathological results. This information was primarily extracted from the electronic medical records.

### 2.6 Statistical analysis

Missing data were imputed using multiple imputation (MI, R package MICE, R Foundation for Statistical Computing, Vienna, Austria, https://cran.r-project.org/, with 20 imputed datasets). This method incorporates randomness in the imputation process to account for the uncertainty of generated values.

Based on expert knowledge, this study primarily investigated the impact of surgical methods on clinical outcomes. The covariates included sex, age, BMI, diabetes status, hypertension status, pancreatic duct diameter, diagnosis (pathological type), and coronary stent placement. The dependent variables included primary and secondary outcomes, with the primary outcome being pancreatic fistula. The secondary outcomes included operative time, postoperative hospital stay, intraoperative blood loss, pancreatic fistula grades A, B, and C rate, severe pancreatic fistula (grades B and C) rate, bile leakage rate, postoperative haemorrhage rate, and perioperative mortality rate.

Quantitative data (age, BMI, pancreatic duct diameter, operative time, intraoperative blood loss, postoperative hospital stay) were compared between the experimental and control groups. Initially, a Kolmogorov-Smirnov (KS) test was used for normality assessment, and an F test was used for homogeneity of variance. For normally distributed data, the mean ± SD was used for description. If variance homogeneity was present, a t test was used for group comparisons; otherwise, a rank-sum test was employed. For nonnormally distributed data in at least one group, the median ± range was calculated, and comparisons were made using a rank-sum test for two completely independent samples. Categorical variables are presented as counts and percentages, with comparisons between test and control groups made using the chi-square test or Fisher’s exact test. The odds ratio (OR) and 95% confidence interval (*CI*) of categorical variables were calculated using two-tailed tests.

For subgroup analysis, pancreatic duct diameter was used for stratification to assess the impact of surgical method on pancreatic fistula in populations with duct diameters <= 3 mm and > 3 mm. Additional assessments were made across different age groups (<60 years, >=60 years), BMI categories (<18.5, 18.5-24.0, >24 kg/m^2^), sex, hypertension status, diabetes status, and stent placement, as well as among different pathological types.

Multiple models have been developed to assess the impact of surgical methods on pancreatic fistula development via sensitivity analysis. The base model (Model 1) included only the surgical method. Model 2 was adjusted for sex, age, and BMI, while Model 3 was further adjusted for diabetes status, hypertension status, pathological type, coronary stent placement, and pancreatic duct diameter. All models were constructed using multivariate logistic regression with all variables included.

All the statistical analyses were performed using R software (version 4.2.3, R Foundation for Statistical Computing, Vienna, Austria, https://cran.r-project.org/). All tests were two-tailed, with a significance level set at *P*<0.05.

## 3 Results

### 3.1 Patient clinical baseline characteristics

Initially, quantitative indicators (age, BMI, pancreatic duct diameter, operative time, postoperative hospital stay, and intraoperative blood loss) of patients in both groups were subjected to normality testing. Age and BMI were found to follow a normal distribution and were thus described using the mean ± standard deviation. In contrast, indicators such as pancreatic duct diameter, operative time, postoperative hospital stay, and intraoperative blood loss did not conform to a normal distribution and were therefore described using the median ± range (**Supplementary Table S1** in **Supplementary 1**).

The study included a total of 78 patients; 43 patients were treated with the five-needle method (test group), and 35 patients were treated with the duct-to-mucosa method (control group). The average age was 63 ± 9 years, with no significant difference in age between the test group and control group (Z=0.116, p=0.908). There were 44 male patients (56.4%). The average BMI for all subjects was 24.7 ± 4.0 kg/m^2^, and the average pancreatic duct diameter was 4 ± 5 mm. Among all the subjects, 28 (35.9%) had pancreatic head cancer, 32 (41.0%) had cholangiocarcinoma, and 18 (23.1%) had duodenal papillary cancer. A total of 33 patients (42.3%) had hypertension, 18 (23.1%) had diabetes, and 7 (9.0%) had undergone coronary stent placement. There were no significant differences in clinical baseline data, such as age, sex, BMI, pancreatic duct diameter, diagnosis, or underlying disease status, between the test group and control group (**Table 2**).

**Table 2:** Comparison of baseline characteristics between the ‘Five-Needle’ method group and the ‘Duct-to-Mucosa’ method group.

Variables	Total n=78	‘Duct-to-Mucosa’ method group n=35	‘Five-Needle’ method Group n=43	t/z/χ^2^	*P*
Age (years, mean ± SD)	63 (9)	63 (9)	63 (10)	-0.116	0.908
<60 years (n(%))	26(33.3)	11(31.4)	15(34.9)	0.104	0.747
>= 60 years(n(%))	52(66.7)	24(68.6)	28(65.1)		
BMI (kg/m^2^, mean ± SD)	24.7(4.0)	24.8 (4)	24.7(4.1)	-0.17	0.865
18.5-24.0(normal, n(%))	25(32.1)	10(28.6)	15(34.9)	0.676	0.713
<18.5 (underweight, n(%))	7(9.0)	4(11.4)	3(7.0)		
>24(overweight, n(%))	46(59.0)	21(60.1)	25(58.1)		
Pancreatic duct diameter (mm, median ± range)	4 (5)	4 (4)	3 (3)	-2.059	0.039
Sex					
Male(n(%))	44(56.4)	20(57.1)	24(55.9)	0.014	0.906
Female(n(%))	34(43.6)	15(42.9)	19(44.2)		
Hypertension					
No(n(%))	45(57.7)	21(60.0)	24(55.8)	0.139	0.71
Yes(n(%))	33(42.3)	14(40.0)	19(44.2)		
Diabetes					
No(n(%))	60(76.9)	27(77.1)	33(76.7)	0.002	0.967
Yes(n(%))	18(23.1)	8(22.9)	10(23.3)		
Coronary stent placement					
No(n(%))	71(91.0)	32(91.4)	39(90.7)	–	0.998
Yes(n(%))	7(9.0)	3(8.6)	4(9.3)		
Pathological type					
Cholangiocarcinoma (n(%))	32(41.0)	16(45.7)	16(37.2)	2.861	0.329
Duodenal papillary cancer (n(%))	18(23.1)	5(14.3)	13(30.2)		
Pancreatic head cancer (n(%))	28(35.9)	14(40.0)	14(32.5)		

### 3.2 Evaluation of clinical outcome indicators

#### 3.2.1 Primary outcome indicators

Twenty-one out of the 78 patients experienced a pancreatic fistula, representing 26.9% of all patients, including 15 with Grade A fistulas (19.2%) and 6 with severe fistulas (grades B and C), 7.7%). In the test group of 43 patients who were treated with the five-needle method, 11 (25.6%) developed a pancreatic fistula (9 Grade A fistulas, 20.9%; 2 severe fistulas, 4.7%), whereas in the control group of 35 patients who were treated with the Duct-to-Mucosa method, 10 (28.6%) developed a pancreatic fistula (6 Grade A fistulas, 17.1%). 4 severe fistulas, 11.4%). There was no significant difference in the occurrence rate of pancreatic fistulas between the test and control groups (25.6% vs. 28.6%, p=0.767). **Table 3**). However, there was a trend towards a lower incidence of pancreatic fistulas in the test group than in the control group (rate difference of 2.99%, 95% CI: -17.75%, 24.32%). Similarly, the percentage of patients with severe pancreatic fistulas tended to be lower in the test group than in the control group (4.7% vs. 11.4%, p=0.400; difference of 6.78%, 95% CI: -6.04, 21.69).

**Table 3:** Comparison of primary and secondary outcome variables between the five-needle method group and the Duct-to-Mucosa method group.

Outcomes	Variable	Total n=78	‘Duct-to-Mucosa’ method group n=35	‘Five-Needle’ method Group n=43	z/χ^2^	P	OR(95% CI)
Primary Outcome	PF						
	No(n(%))	57(73.1)	25(71.4)	32(74.4)	0.088	0.767	0.921 (0.539, 1.574)
	Yes(n(%))	21(26.9)	10(28.6)	11(25.6)			
Secondary Outcomes	PF-A						
	No(n(%))	63(80.8)	29(82.9)	34(79.1)	0.178	0.673	1.151 (0.586, 2.260)
	Yes(n(%))	15(19.2)	6(17.1)	9(20.9)			
	PF-B						
	No(n(%))	74(94.9)	32(91.4)	42(97.1)	–	0.321	0.577 (0.309, 1.075)
	Yes(n(%))	4(5.1)	3(8.6)	1(2.3)			
	PF-C						
	No(n(%))	76(97.4)	34(97.1)	42(97.7)	–	0.998	0.895 (0.219, 3.658)
	Yes(n(%))	2(2.6)	1(2.9)	1(2.3)			
	PF(B+C)						
	No(n(%))	72(92.3)	31(88.6)	41(95.3)	–	0.401	0.646 (0.346, 1.207)
	Yes(n(%))	6(7.7)	4(11.4)	2(4.7)			
	OT(minutes, median ± range)	280 (270)	300 (210)	270 (170)	-2.336	0.019	–
	PHS (days, median ± range)	11 (16)	11 (16)	11 (11)	-0.714	0.475	
	IB(millilitre, median ± range)	150 (300)	150 (250)	150 (250)	-1.331	0.183	
	BL						
	No(n(%))	68(87.2)	30(85.7)	38(88.4)			
	Yes(n(%))	10(12.8)	5(14.3)	5(11.6)	–	0.745	0.882 (0.449, 1.733)
	PH						
	No(n(%))	71(91.0)	32(91.4)	39(90.7)	–	0.998	1.052 (0.431, 2.569)
	Yes (n(%))	7(9.0)	3(8.6)	4(9.3)			
	PD						
	No (n(%))	75(96.2)	33(94.3)	42(97.7)	–	0.999	0.661 (0.285, 1.529)
	Yes(n(%))	3(3.8)	2(5.7)	1(2.3)			

PF, pancreatic fistula; OT, operative time; PHS, postoperative hospital stay; IBL, intraoperative blood loss; BL, bile leak; PH, postoperative haemorrhage; PD, postoperative death.

#### 3.2.2 Secondary outcome indicators

There were no significant differences in the Grade A, Grade B or Grade C pancreatic fistula rate, postoperative hospital stay, intraoperative blood loss, postoperative haemorrhage rate, or postoperative death rate between the test group and the control group (**Table 3**). However, the test group had a significantly shorter operative time (270 ± 170 mins vs. 300 ± 210 mins, p=0.019).

### 3.3 Subgroup analysis

All patients were stratified into groups based on age, BMI, sex, hypertension status, coronary heart disease status, diabetes status, or whether the pancreatic duct diameter exceeded 3 mm. The study revealed that in patients with a pancreatic duct diameter of 3 mm or less (including 3 mm), the incidence of pancreatic fistula in the test group was lower than that in the control group (12.5% vs. 53.8%, p=0.007). Conversely, in patients with a duct diameter greater than 3 mm, the incidence of pancreatic fistula in the test group was higher than that in the control group (41.2% vs. 13.6%, p=0.041). Finally, there were no significant differences in the incidence of pancreatic fistula between the test and control groups across different age groups, sexes, or patients with or without various basic diseases (**Table 4**).

**Table 4:** Subgroup analysis to explore the impact of the characteristics of the ‘Five-Needle’ method group and the ‘Duct-to-Mucosa’ method group.

Variable	Total	‘Duct-to-Mucosa’ method group	‘Five-Needle’ method group	χ2	P	OR(95% CI)
Sex						
male(n,(%))	9(20.5)	4(20.0)	5(20.8)	0.051	0.946	1.029(0.455,2.323)
female(n,(%))	12(34.3)	6(40.0)	6(31.6)	0.261	0.611	0.818(0.384,1.743)
Age						
< 60 years	7(26.9)	3(27.3)	4(26.7)	0.001	0.973	0.982(0.360,2.684)
>= 60 years	38(26.9)	7(29.2)	7(25.0)	0.114	0.763	0.895(0.476,1.683)
BMI						
18.5-24.0 kg/m^2^ (normal, n(%))	5(20.0)	3(30.0)	2(13.2)	1.041	0.307	0.583(0.230,1.482)
<18.5 kg/m^2^ (underweight, n(%))	0(0.0)	0(0.0)	0(0.0)	–	–	–
>24 kg/m^2^ (overweight, n(%))	16(34.8)	7(33.3)	9(36.0)	0.036	0.849	1.067(0.543, 2.094)
Hypertension						
No(n(%))	9(20.0)	5(23.8)	4(16.7)	0.357	0.551	0.800(0.402, 1.594)
Yes(n(%))	12(36.4)	5(35.7)	7(36.8)	0.004	0.971	1.029(0.448,2.363)
Type 2 diabetes						
No(n(%))	16(26.7)	7(25.9)	9(27.3)	0.014	0.907	1.039(0.546,1.976)
Yes(n(%))	5(27.8)	3(37.5)	2(20.0)	0.678	0.411	0.641(0.238,1.729)
Pathology						
cholangiocarcinoma(n(%))	10(31.3)	5(31.3)	5(31.3)	0.001	0.998	1.000(0.474,2.112)
Carcinoma of duodenal papilla(n(%))	3(16.7)	2(40.0)	1(7.7)	2.714	0.099	0.300(0.083,1.090)
pancreatic cancer(n(%))	8(28.6)	3(21.4)	5(35.7)	0.701	0.403	1.467(0.551,3.902)
Pancreatic duct diameter						
<=3 mm	10(27.0)	7(53.8)	3(12.5)	7.039	0.007	0.317(0.141,0.716)
> 3 mm	11(26.8)	3(13.6)	8(41.2)	4.209	0.041	2.322(0.852,6.329)
Coronary stent implantation						
No(n(%))	17(23.9)	8(25.0)	9(23.1)	0.036	0.851	0.944(0.526,1.697)
Yes(n(%))	4(57.1)	2(66.7)	2(50.0)	0.194	0.659	0.667(0.102,4.354)

### 3.4 Sensitivity analysis

Multivariate logistic regression analysis was used to develop several models. Model 1 included the surgical method as the sole independent variable. Models 2 and 3 incorporated adjustments for various covariates. In all three models, the incidence of pancreatic fistula did not significantly differ between the test and control groups, as indicated by the 95% CI of the OR encompassing 1. However, the regression coefficients were negative regarding the intervention measures and presence of pancreatic fistula. This suggests that the absolute number of pancreatic fistulas was lower in the test group than in the control group, as detailed in **Supplementary Table S2** of **Supplementary 1**.

## 4 Discussion

The pancreas secretes approximately 1-2 litres of pancreatic juice daily in a healthy adult, containing a plethora of digestive enzymes (pancreatic lipase, protease, and amylase) essential for the digestion and absorption of food in the intestine. Postpartial pancreatectomy for benign or malignant pancreatic tumours, or even pancreatic trauma, is often necessary to re-establish an anastomosis between the remaining pancreatic duct and the jejunum to ensure the smooth entry of pancreatic juice into the intestine. However, due to the unique physicochemical properties of pancreatic juice, the activation of its digestive enzymes can degrade sugars, proteins, and fats. In the context of pancreatic surgery, leakage of pancreatic fluid into the peritoneal cavity can lead to severe complications such as infection, haemorrhage, and enteric fistulas, posing a significant risk to the patient’s postoperative survival. Consequently, surgeons continually explore various methods to prevent pancreatic leakage, including improvements in surgical methods, intraoperative protective measures, and perioperative pharmacological prevention. Over the years, the methods for pancreatojejunostomy have evolved from initial pancreatic stump-jejunum invagination and pancreatic stump-gastric invagination to the more commonly used ‘Duct-to-Mucosa’ anastomosis (11, 13), which is widely applied in both laparoscopic and open pancreaticoduodenectomy. Despite these advances, the incidence of postoperative pancreatic fistula and related complications has not significantly decreased, and the occurrence of pancreatic fistula after pancreatojejunostomy is still a major challenge impeding the advancement of pancreatic surgery (14–16).

With the advancement of minimally invasive methods, laparoscopic pancreatic surgery has become increasingly prevalent. However, the flexibility of laparoscopic operations is somewhat limited, and certain anatomical angles are less than ideal, especially during pancreatojejunostomy. The small angle between the needle and the needle holder makes the procedure awkward. Furthermore, in cases where the pancreatic texture is soft and the pancreatic duct diameter is small, traditional anastomosis methods are time-consuming and imprecise, potentially exacerbating serious postoperative complications such as pancreatic fistula. This is particularly true for patients with narrow pancreatic ducts, where suturing is difficult, and the incidence of postoperative pancreatic fistula is relatively high (17, 18). Additionally, the difficulty of performing pancreatojejunostomy is a major reason for a prolonged operation and conversion to open surgery, adversely affecting patient safety during the perioperative period and postoperative recovery, as well as undermining the surgeon’s confidence. In recent years, to adapt to LPD, domestic scholars have made various improvements and proposed more straightforward methods, such as Hong Defei’s “Hong’s One-Needle” method and Liu Rong’s “301” method (7, 8, 19, 20). These methods achieve biological healing through precise apposition of the cut pancreatic surface to the jejunal serosal layer, while pancreatic juice is drained into the jejunal cavity through a stent tube, thereby preventing pancreatic fistula (19). In summary, with the development of minimally invasive methods and surgeons gaining a deeper understanding of the importance of physiological healing at anastomotic sites, simplified and safe pancreatojejunostomy methods are gradually gaining acceptance over previous, more complex methods.

Drawing inspiration and learning from the methods and experiences of several domestic predecessors in laparoscopic and robot-assisted pancreatojejunostomy, the new five-needle method was proposed for laparoscopic pancreatojejunal mucosal anastomosis (as detailed in the surgical methods section). Compared to other anastomosis methods, this method has the following main characteristics. The pancreatic stump is completely sutured using a through-and-through method, eliminating the need for interrupted or continuous suturing from the pancreatic duct to the jejunum. As such, it is not limited by the diameter of the pancreatic duct and is even more suitable for patients with a narrow pancreatic duct, a soft pancreatic texture, and a small pancreatic neck. None of the five sutures are passed through the full thickness of the pancreas and intestine, reducing the impact on the blood supply of the pancreatojejunostomy site and preventing the penetrating injury of sutures to the pancreatic duct and intestinal tube, which could lead to the leakage of pancreatic fluid through the suture holes, forming a fistula. For pancreatojejunostomy, we adhere to the principles of ‘tight, loose, sparse’ (tight apposition between the pancreatic cut surface and the intestinal wall, leaving no dead space; natural stacking of the intestine on the cut surface after being brought up to the wound, ensuring no tension postanastomosis; and sparse suturing to minimize tissue cutting injury and ischaemia). This method aligns well with these principles and robustly ensures the safety of the anastomosis site.

In our study, the clinical data of 78 patients were collected to investigate the efficacy of the five-needle method for pancreaticojejunostomy during laparoscopic pancreaticoduodenectomy. The findings revealed that the method did not significantly differ from the traditional ‘Duct-to-Mucosa’ method, particularly in terms of the incidence of pancreatic fistula, the incidence of biliary leakage, the rate of postoperative haemorrhage, the rate of perioperative mortality, the duration of hospital stay, or the intraoperative blood loss volume. Further analysis through multivariable logistic regression revealed negative regression coefficients across the three models, indicating a lower absolute incidence of pancreatic fistula in the experimental group than in the control group. The consistency of the results across different models underscores their robustness. This signifies that the ‘Five-Needle’ method for pancreaticojejunostomy is not only safe and effective but also has a significantly lower incidence of clinical fistula than the control group, making it a promising method worthy of broader clinical application.

According to the secondary observational indices, the test group had significant advantages over the control group in certain aspects. First, statistically, the overall operative time for the conventional anastomosis method was significantly longer than that for the five-needle method. Since this was a retrospective study, we did not have data on the duration of pancreato-enterostomy. However, as an important surgical step, it was believed that the reduction was attributable to the decrease in pancreaticojejunostomy time. The primary aim of the five-needle method was to simplify the anastomosis process, thereby reducing the operative time, minimizing the need for conversion to open surgery, and enhancing confidence among novice surgeons. Second, as previously mentioned, the diameter of the pancreatic duct is a crucial objective factor influencing pancreaticojejunostomy, especially in cases of narrower ducts where anastomosis is relatively challenging. In our study, there was no statistically significant difference in the incidence of pancreatic fistula between the two surgical methods. However, through subgroup analysis of pancreatic duct diameter, it was found that in patients with a duct diameter of 3 mm or less, the incidence of pancreatic fistula in the five-needle method group was lower than that in the duct-to-mucosa group. This further confirms the suitability of the ‘Five-Needle’ method for patients with narrower pancreatic ducts, as it has high practical value in clinical practice. The advantage arises from our focus on ensuring closer alignment between the support tube and the pancreatic duct during suturing, without the need to ensure that the stitches precisely penetrate the pancreatic duct.

There are still many shortcomings in the study. First, although there was no significant difference between the two groups in the statistical analysis of baseline data in this study, the selection of surgical methods is susceptible to interference by patients’ objective conditions because this was a retrospective study. Second, the collection of observation indicators, such as the amount of abdominal drainage fluid and the drainage time of pancreatic fistula patients, was not detailed enough, and the duration of pancreatoenteroanastomosis was also not available. Third, the study was an exploratory study, with a small sample size that does not allow for definitive conclusions. Fourth, in the realm of clinical predictive modelling research, nonlinear analysis has progressively become a focal method, and multitemporal data hold greater value (21, 22). Therefore, in future predictive studies, the incorporation of multidimensional and multitemporal models, as well as nonlinear models, should be duly integrated into the endeavours undertaken at our research centre. In addition, there is a need for larger scale, multicentre randomized, and open studies to establish more robust findings in the future.

## 5 Conclusion

In summary, the five-needle method can significantly reduce the incidence of pancreatic fistula and shorten the operative time without increasing the incidence of other surgery-related complications in patients with narrower pancreatic ducts and is a safe and effective laparoscopic method for LPD. Therefore, the application of this new method can further promote the widespread adoption of LPD, warranting its broader clinical implementation.

## Data availability statement

The raw data supporting the conclusions of this article will be made available by the authors, without undue reservation.

## Author contributions

ZW: Conceptualization, Methodology, Writing – original draft. BZ: Investigation, Project administration, Resources, Validation, Writing – review & editing. HX: Data curation, Formal analysis, Supervision, Visualization, Writing – original draft, Writing – review & editing. WZ: Conceptualization, Investigation, Methodology, Project administration, Resources, Writing – review & editing.
