# Supplementary material for: Efficacy of the 'Five-Needle' method for pancreatojejunostomy in laparoscopic pancreaticoduodenectomy: an observational study
Source: Front Oncol. 2024 Apr 16;14:1347752. doi: 10.3389/fonc.2024.1347752 (PMC11058832; doi:10.3389/fonc.2024.1347752)
Supplement: Supplementary file 1 [file DataSheet_1.docx]

1. Ma CX, Gao F, Luo J, Northfelt DW, Goetz M, Forero A, et al. NeoPalAna: neoadjuvant palbociclib, a cyclin-dependent kinase 4/6 inhibitor, and anastrozole for clinical stage 2 or 3 estrogen receptor-positive breast cancer. *Clin Cancer Res* (2017), 23, 4055–4065, doi: 10.1158/1078-0432.CCR-16-3206
2. Johnston S, Puhalla S, Wheatley D, Ring A, Barry P, Holcombe C, et al. Randomized phase II study evaluating palbociclib in addition to letrozole as neoadjuvant therapy in estrogen receptor-positive early breast cancer: PALLET trial. *J Clin Oncol* (2019), 37, 178–189. doi: 10.1200/JCO.18.01624.
3. Chow LWC, Morita S, Chow CYC, Ng W-K, Toi M. Neoadjuvant palbociclib on ER+ breast cancer (N007): clinical response and EndoPredict's value. *Endocr Relat Cancer* (2018), 25, 123-130. doi: 10.1530/ERC-17-0396.
4. Cottu P, D’Hondt V, Dureau S, Lerebours F, Desmoulins I, Heudel P-E, et al. Letrozole and palbociclib versus chemotherapy as neoadjuvant therapy of high-risk luminal breast cancer. *Ann Oncol* (2018), 29, 2334–2340, doi:10.1093/annonc/mdy448
5. Curigliano G, Pardo PG, Meric-Bernstam F, Conte P, Lolkema MP, Beck JT, et al. Ribociclib plus letrozole in early breast cancer: a presurgical, window-of-opportunity study. *Breast* (2016), 28, 191–198, doi: 10.1016/j.breast.2016.06.008.
6. Hurvitz SA, Martin M, Press M.F, Chan D, Fernandez-Abad M, Petru E, et al. Potent Cell-Cycle Inhibition and Upregulation of Immune Response with Abemaciclib and Anastrozole in neoMONARCH, Phase II Neoadjuvant Study in HR+/HER2- Breast Cancer. *Clin Cancer Res* (2020), 26, 566-580. doi: 10.1158/1078-0432.CCR-19-1425.
7. Khan QJ, O'Dea A, Bardia A, Kalinsky K, Wisinski KB, O'Regan R, et al. Letrozole + ribociclib versus letrozole + placebo as neoadjuvant therapy for ER+ breast cancer (FELINE trial). *J Clin Oncol* (2020), 38, no. 15_suppl 505-505.DOI: 10.1200/JCO.2020.38.15_suppl.505
