# Supplementary material for: Efficacy of the 'Five-Needle' method for pancreatojejunostomy in laparoscopic pancreaticoduodenectomy: an observational study
Source: Front Oncol. 2024 Apr 16;14:1347752. doi: 10.3389/fonc.2024.1347752 (PMC11058832; doi:10.3389/fonc.2024.1347752)
Supplement: Supplementary file 2 [file Table_1.docx]

**Table 1. Neoadjuvant studies with CDK 4/6 inhibitors with published results.**

| **Study** | **Phase** | **Treatments** | **Patients enrolled** | **Primary Study objectives** | **Secondary Study objectives** | **Results** | **Ref** |
| --- | --- | --- | --- | --- | --- | --- | --- |
| NeoPalAna | II | Palbo+anastrozolo | 50 | (CCCA: central Ki67 2.7%) | CCCA and Ki67 response by baseline PAM50-based intrinsic subtypes, clinical, radiological, and pathologic response, safety profiles | CCCA rate C1D15 87% vs.  C1D1 26%, (P < 0.001)  Response rate: 80% (clinical), 41% (US), 52% (RX)  pCR:0 | 16 |
| Pallet | Rand II | Letrozole 14 wks(a)  Letrozole 2 wks🡪  Palbo plus letrozole 14 wks(b)  palbo 2 wks🡪palbo+letrozole©  palbo+letrozole (d) | 307 | change in Ki-67 | clinical response (ordinal and ultrasound) after 14 weeks | Clinical response not significantly different  Greater change in ki67 by combo | 17 |
| N007 | Single-arm | palbo plus letrozole | 20 | cRR and  (PEPI) | pathologic response  and gene expression testing with EP test | Clinical response: 85%  US-response: 70%  pCR: 5% | 18 |
| LETPAL | Rand II | palbo plus letrozole (a)  chemotherapy (b) | 106 | RCB | clinical response, proliferation-based markers, safety | RCB 0-I  7.7% (a) vs 15.7% (b)  similar cRR  pCR 3.7% (a) vs 5.6% (b) | 19 |
| Monaleesa-I | Rand II | Ribo plus letrozole (a)  Letrozole 400 mg, 600 mg (b,c) | 14 | Ki67 change | safety, PK (a), PD markers (b) | Ki67 reduction in b and c> a | 20 |
| NeoMonarch | Rand II | 2 wks lead-in abema (a), anastrozole(b), abeam+anastrozole(c)🡪14 wka abema+anastrozole | 224 | Ki67 change | clinical, radiologic, and pathologic responses,  safety, gene expression changes | In lead-in phase abema induces>decrease ki67.  ORR 54%, pCR 4%  Radiological ORR 46%  pCR:4% | 21 |
| Feline | Rand II | Letrozole+placebo  Letrozole+ribociclib | 120 | PEPI | Ki-67, CCCA: Ki-67 <2.7%, clinical/imaging response, and difference in response & toxicity | No difference | 22 |

**Figure legend**: Rand: randomized; Palbo:palbociclib; Ribo: ribociclib; abema: abemaciclib; CCCA: complete cell cycle arrest ; cRR: clinical response rate; PEPI: preoperative endocrine prognostic index; EP: EndoPredict; RCB: residual cancer burden; PD: pharmacodynamic; pCR: Pathological complete response.
